# Comparative Survival Analysis of Anti‐Angiogenic Agent Plus Immunochemotherapy in NSCLC Patients After Frontline EGFR‐TKI Treatment: A Retrospective Cohort Study

**DOI:** 10.1002/kjm2.70023

**Published:** 2025-04-28

**Authors:** Yi‐Tse Su, Shu‐Farn Tey, Chung‐Ta Lee, Chien‐Yu Lin, Jeng‐Shiuan Tsai, Chien‐Chung Lin, Chin‐Wei Kuo

**Affiliations:** ^1^ Division of Chest Medicine, Department of Internal Medicine, National Cheng Kung University Hospital, College of Medicine National Cheng Kung University Tainan Taiwan; ^2^ Division of Pulmonary Medicine Chi‐Mei Medical Center Tainan Taiwan; ^3^ School of Medicine, College of Medicine National Sun Yat‐Sen University Kaohsiung Taiwan; ^4^ Department of Pathology, College of Medicine National Cheng Kung University Tainan Taiwan

**Keywords:** anti‐angiogenic agent, EGFR mutation, immunotherapy, NSCLC, survival

## Abstract

Advanced‐stage EGFR‐mutated lung non‐small cell lung cancer (NSCLC) challenges current treatment paradigms, particularly after frontline EGFR‐TKI therapy failure. This study investigates the survival impact of combined anti‐angiogenic agent and immunochemotherapy (AICT) for this population. We retrospectively analyzed NSCLC patients at National Cheng Kung University Hospital from January 2010 to December 2022, focusing on those who had disease progression beyond frontline EGFR‐TKI treatments. Survival outcomes were assessed through progression‐free survival (PFS) and overall survival post‐TKI failure (OSpTKI). Propensity score was employed to match patients, with Kaplan–Meier curve and multivariable Cox regression analysis determining the survival benefits. Analyses were also performed for subgroups based on PD‐L1 level, treatment lines, and regimens. A total of 412 patients were enrolled, with 27 receiving AICT. Compared to patients who did not receive AICT, those who received AICT had longer PFS (5.9 vs. 3.9 months, *p* = 0.024) and longer OSpTKI (17.9 vs. 11.9 months, *p* = 0.018). The observed survival advantage in PFS and OSpTKI was consistent in both the original cohort (for PFS: hazard ratio [HR] = 0.59, 95% confidence interval [CI] = 0.39–0.90, *p* = 0.014; for OSpTKI: HR = 0.41, 95% CI = 0.24–0.69, *p* < 0.001) and after propensity score matching (for PFS: HR = 0.56, 95% CI = 0.35–0.98, *p* = 0.014; for OSpTKI: HR = 0.45, 95% CI = 0.26–0.79, *p* = 0.006). In the subgroup analyses, patients with PD‐L1 ≥ 1%, those who received AICT as a second‐line therapy, or those treated in conjunction with pemetrexed showed a PFS benefit. AICT improves survival outcomes in advanced‐stage EGFR‐mutated NSCLC, advocating for its integration into treatment regimens.

## 1 Introduction

Despite advancements in treatments for metastatic non‐small cell lung cancer (NSCLC), patients harboring sensitizing epidermal growth factor receptor (EGFR) mutations ultimately experience treatment failure with frontline tyrosine kinase inhibitors (TKIs) [1, 2]. Although immune checkpoint inhibitors, such as anti‐programmed death ligand 1 (PD‐L1) or PD‐1 agents, have yielded improved survival for NSCLC patients lacking sensitizing driver mutations [3, 4], their use as initial therapy for patients with sensitizing EGFR mutations is not recommended due to lack of effectiveness [5, 6, 7]. Recent Phase 3 clinical trials CheckMate 772 and Keynote 789, which investigated the combination of nivolumab or pembrolizumab with chemotherapy, failed to demonstrate a survival benefit in the second‐line setting after failure of first‐line EGFR‐TKI [8, 9].

On the other hand, the combination of immunotherapy, chemotherapy, and anti‐angiogenic agents may offer survival benefits for patients with sensitizing EGFR mutations following the frontline TKI. Specifically, a post hoc analysis of the IMpower150 study highlighted that atezolizumab in combination with bevacizumab, carboplatin, and paclitaxel (ABCP) exhibited improved overall survival (OS) compared to the group treated with bevacizumab, carboplatin, and paclitaxel (BCP) among patients with sensitizing EGFR mutations [10]. The ATTLAS study assessed the efficacy of ABCP, followed by maintenance atezolizumab and bevacizumab, against platinum doublet chemotherapy followed by maintenance pemetrexed in EGFR‐ or ALK‐mutated NSCLC after TKI progression. It found improved progression‐free survival (PFS) with increased PD‐L1 levels; however, it noted no significant improvement in OS [11]. Additionally, the ORIENT‐31 Phase 3 trial, assessing a novel anti‐angiogenesis and immunochemotherapy regimen including sintilimab, IBI305, cisplatin, and pemetrexed for non‐squamous NSCLC patients post‐EGFR‐TKI therapy progression, reported a persistent PFS advantage in its interim findings [12]. Nonetheless, beyond these randomized control trials, literature on this subject, even including retrospective studies, remains scarce and presents conflicting results, particularly regarding OS [13, 14, 15, 16].

The conflicting results from recent trials highlight a clear gap in the understanding of the effectiveness of combining anti‐angiogenic agent, immunotherapy, with chemotherapy (AICT) for these EGFR mutant NSCLC patients who had disease progression after frontline EGFR‐TKI treatment. Therefore, we initiated a retrospective cohort study to evaluate the survival benefits of AICT in patients with advanced‐stage EGFR‐mutated NSCLC following progression on frontline EGFR‐TKI treatment.

## 2 Methods

### 2.1 Study Design and Patient Enrollment

This study received approval from the Institutional Review Board of National Cheng Kung University Hospital (NCKUH) prior to commencement (IRB number: B‐ER‐112‐118). We retrospectively enrolled patients with advanced‐stage NSCLC harboring EGFR mutations who received first‐line EGFR‐TKI treatment from January 1, 2010, to December 31, 2022. We collected baseline characteristics such as age, sex, Eastern Cooperative Oncology Group performance status (ECOG‐PS), cancer histology subtype, initially diagnosed stage, site of distant metastasis, subtypes of EGFR mutation, cancer treatments across different lines, disease status, and survival. These data were extracted from the electronic medical record system of NCKUH. The cutoff date for survival status follow‐up was March 30, 2024. PD‐L1 levels were recorded for patients who received AICT, but we did not record PD‐L1 levels for patients who did not receive AICT due to a high proportion of missing data. This is because our hospital did not routinely assess PD‐L1 levels in patients with EGFR mutations unless immunotherapy was being considered. In this investigation of survival benefits following frontline EGFR‐TKI treatment, we excluded patients who exhibited no disease progression, who did not receive subsequent cancer treatment after the frontline EGFR‐TKI, or who were lost to follow‐up at NCKUH. Additionally, to mitigate imbalance in first‐line treatment outcomes, patients who received third‐generation EGFR‐TKIs as their first‐line treatment for NSCLC were also excluded.

### 2.2 Surveillance Strategy and Outcomes Definition

Patients were scheduled for chest computed tomography scans every 12 weeks to evaluate tumor responses. Brain magnetic resonance imaging and whole‐body bone scan were arranged based on clinical symptoms after beginning treatment. OS was defined as the interval from the initiation of treatment to the date of death. PFS was calculated from the treatment start date to the occurrence of death or radiological progression, in accordance with the Response Evaluation Criteria in Solid Tumors (RECIST) version 1.1 [17]. For participants who neither progressed nor died, data were censored on the last follow‐up date (March 30, 2024). The study's endpoints included PFS following the initiation of treatment after frontline EGFR‐TKI therapy (PFSpTKI), PFS with anti‐angiogenic agent plus immunochemotherapy (PFSwAICT) and OS post‐frontline TKI therapy (OSpTKI) (Figure S1). Given the limited cases in which AICT was administered as the initial therapy following frontline TKI, our study compared PFSwAICT in patients treated with AICT against PFSpTKI in those who were not treated with AICT. For clarity, we uniformly refer to this measure as “PFS” rather than using separate terms such as PFSwAICT or PFSpTKI.

### 2.3 Statistical Analysis

Data were summarized as counts (percentages), means (standard deviations [SD]), or medians (interquartile ranges [IQRs]), depending on the characteristics. The Mann–Whitney *U* test or the independent samples *t*‐test was employed to assess continuous variables, contingent upon their distribution's adherence to normality. Comparisons of categorical variables were executed using Fisher's exact test. The Kaplan–Meier method was utilized to evaluate differences in PFS and OSpTKI. We conducted multivariable Cox proportional hazards regression analyses to investigate the potential benefits on PFS and OSpTKI associated with the use of AICT. The analysis adjusted for a variety of covariates, including age, gender, Eastern Cooperative Oncology Group performance status (ECOG‐PS), stage of cancer, and cancer type, in addition to the presence of metastasis and type of first‐line EGFR‐TKI treatment. To quantify the strength and direction of these associations, we estimated hazard ratios (HRs) along with their 95% confidence intervals (CIs). To verify the survival advantage of AICT across different PD‐L1 levels, treatment lines, and chemoimmunotherapy combinations, we undertook additional subgroup analyses. Patients were categorized by PD‐L1 level, the treatment line of AICT initiation following initial TKI treatment, and different chemoimmunotherapy plus bevacizumab combinations. To bolster the validity of our survival analysis results and reduce the influence of confounding factors, we additionally conducted analyses incorporating propensity score matching. Propensity scores were derived using a logistic regression model that incorporated the confounders previously specified in the Cox proportional hazards regression analysis. Subsequently, a 1:5 matching strategy was applied to compare the groups receiving AICT and those not receiving AICT. The balance between variables was assessed using the absolute standardized mean difference (ASMD), with an ASMD of less than 0.25 indicating satisfactory balance, as suggested by previous research [18]. All statistical tests conducted were two‐sided, considering *p* values less than 0.05 as indicative of statistical significance. These analyses were executed using the SAS software, version 9.4 (SAS Institute, Cary, NC, USA).

## 3 Results

### 3.1 Patient Characteristics for Enrolled Patients

**FIGURE 1:**
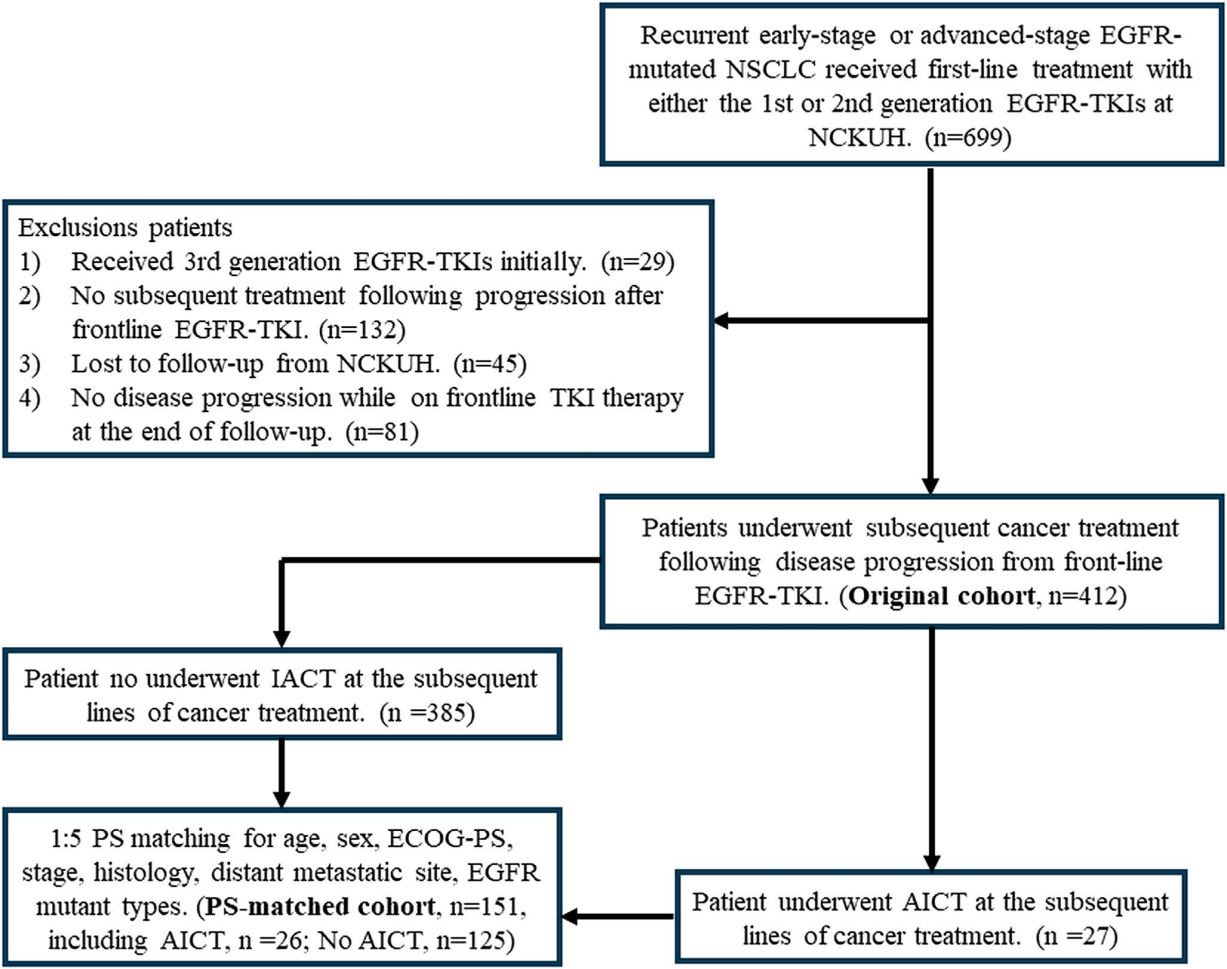
Algorithm for patient enrollment and PS matching. AICT, anti‐angiogenic agent plus immunochemotherapy; ECOG‐PS, Eastern Cooperative Oncology Group performance score; EGFR, epidermal growth factor receptor; NCKUH, National Cheng Kung University Hospital; NSCLC, non‐small cell lung cancer; PS, propensity score; TKI, tyrosine kinase inhibitor.

Between January 2010 and December 2022, there was a total of 699 patients diagnosed with advanced‐stage EGFR‐mutated NSCLC who received first‐line treatment with either the first‐ or second‐generation EGFR‐TKIs at NCKUH. Exclusions were made for 29 patients who received third‐generation TKIs as their initial treatment, 132 patients who did not undergo subsequent treatment following progression after frontline TKI therapy, 45 patients who were lost to follow‐up from NCKUH, and 81 patients who showed no disease progression while on frontline TKI therapy. Consequently, the analysis included 412 patients, of whom 27 had received AICT and 385 had not received AICT (Figure 1). Patients who received AICT were generally younger, with a median age of 58.8 years compared to 63.3 years in those who did not receive AICT (*p* = 0.038). A higher prevalence of exon 19 deletions was observed among AICT recipients (70.4% vs. 44.2%, *p* = 0.009), while the presence of the L858R mutation was less common (22.2% vs. 49.6%, *p* = 0.009) (Table 1). There were no statistically significant differences between the two groups regarding gender, cancer histology, stage of cancer, sites of distant metastasis, and types of first‐line EGFR‐TKIs. For the AICT group, one patient did not receive PD‐L1 level testing. A total of 12 patients (44.4%) had a PD‐L1 expression level of ≥ 1%, and none of the patients had a PD‐L1 ≥ 50%. Compared to patients who did not receive AICT, those who ever received AICT were more likely to undergo therapy with immunotherapy (70.4% vs. 1.2%, *p* < 0.001) and anti‐angiogenic agents (70.4% vs. 2.7%, *p* < 0.001) following failure of frontline EGFR‐TKI treatment as the initially subsequent treatment.

**TABLE 1:** Baseline characteristics of all enrolled patients.

Characteristics	Original cohort (*n* = 412)			PS‐matched cohort (*n* = 151)		
	AICT (*n* = 27)	No AICT (*n* = 385)	*p* ^a^	AICT (*n* = 26)	No AICT (*n* = 125)	ASMD
Age (years), mean (SD)	58.8 (11.8)	63.3 (10.9)	0.038	59.9 (10.5)	59.5 (10.9)	0.003
Male, *n* (%)	12 (44.4)	150 (39.0)	0.684	11 (42.3)	49 (39.2)	0.063
ECOG‐PS ≥ 1, *n* (%)	11 (40.7)	134 (34.8)	0.537	10 (38.5)	51 (40.8)	0.048
Histology, *n* (%)			0.626			
Adenocarcinoma	27 (100)	366 (95.1)		26 (100)	125 (100)	< 0.001
Others	0 (0)	19 (4.9)		0 (0)	0 (0)	< 0.001
Stage, *n* (%)			0.346			
III	2 (7.4)	20 (5.2)	0.647	2 (7.7)	9 (7.2)	0.020
IV	25 (92.5)	365 (94.8)	0.647	24 (92.3)	116 (92.8)	0.020
EGFR mutation subtypes, *n* (%)						
Exon 19 deletion	19 (70.4)	170 (44.2)	0.009	18 (69.2)	82 (65.6)	0.076
L858R	6 (22.2)	191 (49.6)	0.009	6 (23.1)	36 (28.8)	0.124
Others	2 (7.4)	24 (6.2)		2 (7.7)	7 (5.6)	
PD‐L1 level						
≥ 1%	12 (44.4)	—		11 (42.3)	—	
< 1%	14 (51.9)	—		14 (53.8)	—	
Not detected	1 (11.1)	—		1 (11.5)	—	
Distant metastatic site, *n* (%)						
Brain	10 (37.0)	104 (27.0)	0.270	10 (38.5)	42 (33.6)	0.105
Pleura	11 (40.7)	194 (50.4)	0.427	11 (42.3)	51 (40.8)	0.030
Liver	2 (7.4)	45 (11.7)	0.755	2 (7.7)	10 (8.0)	0.011
First‐line EGFR‐TKI, *n* (%)			0.072			
First generation	15 (55.6)	283 (73.5)		15 (57.7)	93 (74.4)	0.371
Second generation	12 (44.4)	102 (26.5)		11 (42.3)	32 (25.6)	0.371
Subsequent treatment after EGFR‐TKI, *n* (%)						
Chemotherapy	26 (96.3)	343 (83.3)	0.339	26 (100.0)	110 (88.0)	
Immunotherapy	19 (70.4)	5 (1.2)	< 0.001	19 (73.1)	1 (0.8)	
Anti‐angiogenesis	19 (70.4)	11 (2.7)	< 0.001	19 (73.1)	4 (3.2)	
Other	1 (3.7)	40 (9.7)	0.501	0 (0)	14 (11.2)	
AICT regimen						
Pem, atezo plus anti‐angiogenesis	15 (55.6)	—		14 (53.9)	—	
Tax/gem, atezo plus anti‐angiogenesis	10 (37.0)	—		10 (38.5)	—	
Other IO, CT plus anti‐angiogenesis	2 (7.4)	—		2 (7.7)	—	
AICT Treatment lines, *n* (%)						
2 L	17 (63.0)	—		17 (65.4)	—	
> 2 L	10 (37.0)	—		9 (34.6)	—	

Abbreviations: AICT, anti‐angiogenic agent plus immunochemotherapy; ASMD, absolutely standardized mean difference; atezo, atezolizumab; CT, chemotherapy; ECOG‐PS, Eastern Cooperative Oncology Group performance score; EGFR, epidermal growth factor receptor; Gem, gemcitabine; IO, immunotherapy; PD‐L1, programmed cell death ligand 1; Pem, pemetrexed; PFS, progression‐free survival; PS, propensity score; SD, standard deviation; Tax, taxanes; TKI, tyrosine kinase inhibitor.

^a^ Independent *t*‐test, Mann–Whitney *U* test, and Fisher exact test were used to calculate continuous variables and categorical variables, respectively.

Following 1:5 propensity score matching, a cohort of 26 patients who received AICT and 125 patients who did not receive AICT was established. The detailed characteristics of these groups are presented in Table 1. Balance between the groups was achieved for all variables except for the use of first‐generation EGFR‐TKIs. However, this exception did not impact the outcomes of PFSpTKI and OSpTKI.

### 3.2 Survival Analyses for the Use of AICT

**FIGURE 2:**
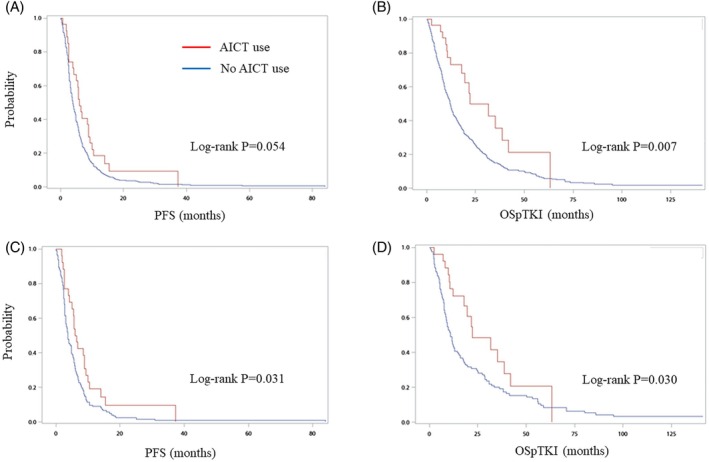
Kaplan–Meier plot and log‐rank test for survival in patients receiving AICT and the control group. For full cohort (A) PFS, (B) OSpTKI. For PS‐matched cohort (C) PFS, (D) OSpTKI. AICT, anti‐angiogenesis plus immunochemotherapy; OSpTKI, overall survival post‐frontline tyrosine kinase inhibitor; PFS, progression‐free survival; PS, propensity score.

In the original cohort, patients who had received AICT exhibited a longer median PFS compared to those who did not receive AICT (5.9 vs. 3.9 months, *p* = 0.024). Furthermore, these patients also demonstrated longer OSpTKI (17.9 vs. 11.9 months, *p* = 0.018) when compared to patients without AICT (Table 2). Kaplan–Meier survival analysis indicated a trend toward extended PFS in patients who received AICT compared to those who did not receive AICT (log‐rank *p* = 0.054). Additionally, patients treated with AICT experienced significantly longer OSpTKI compared to patients who did not receive AICT (Figure 2A,B). Multivariable Cox proportional hazards regression analysis revealed that the utilization of AICT was significantly correlated with a reduced risk of disease progression, with a HR of 0.59 (95% CI = 0.39–0.90, *p* = 0.014). Additionally, AICT use was associated with decreased mortality in OSpTKI (HR = 0.41, 95% CI = 0.24–0.69, *p* < 0.001) (Table 3).

**TABLE 2:** Survival outcomes of the enrolled patients and different subgroups.

Median survival (IQR), months	Original cohort (*n* = 412)			PS‐matched cohort (*n* = 151)		
	AICT	No AICT	*p* ^a^	AICT	No AICT	*p* ^a^
Full cohort	*n* = 27	*n* = 385		*n* = 26	*n* = 125	
PFS	5.9 (7.3)	3.9 (4.6)	0.024	6.2 (6.0)	3.7 (4.6)	0.012
OSpTKI	17.9 (21.1)	11.9 (18.6)	0.018	17.7 (21.1)	10.8 (20.7)	0.033
By PD‐L1						
≥ 1%						
PFS	9.4 (9.0)		0.017	8.8 (3.8)		0.003
OSpTKI	17.7 (12.1)		0.267	17.5 (13.4)		0.307
< 1%						
PFS	5.6 (3.9)		0.331	5.6 (3.9)		0.315
OSpTKI	17.2 (19.5)		0.054	17.2 (19.5)		0.061
By AICT treatment lines						
2 L	*n* = 10			*n* = 9		
PFS	7.8 (7.6)		0.057	8.8 (6.2)		0.013
OSpTKI	17.0 (9.3)		0.114	16.5 (7.3)		0.135
> 2 L	*n* = 17			*n* = 17		
PFS	5.7 (6.2)		0.149	5.7 (6.2)		0.152
OSpTKI	18.0 (21.3)		0.071	18.0 (21.3)		0.096
By AICT regimen						
CT with Pem	*n* = 15			*n* = 14		
PFS	8.8 (9.2)		0.039	8.8 (7.9)		0.011
OSpTKI	17.5 (11.7)		0.253	17.0 (9.6)		0.305
CT with Gem or Tax	*n* = 10			*n* = 10		
PFS	5.6 (3.4)		0.587	5.6 (3.4)		0.594
OSpTKI	23.5 (26.5)		0.036	23.5 (26.5)		0.050
Other IO and CT	*n* = 2			*n* = 2		
PFS	11.0 (9.1)		0.108	11.0 (9.1)		0.114
OSpTKI	36.9 (52.9)		0.309	36.9 (52.9)		0.309
By EGFR mutation						
Exon 19 deletion	*n* = 19	*n* = 170		*n* = 18	*n* = 82	
PFS	5.6 (6.7)	3.9 (4.1)	0.138	5.7 (6.7)	3.7 (4.6)	0.140
OSpTKI	12.4 (11.7)	10.1 (17.2)	0.158	12.3 (9.6)	9.2 (19.1)	0.249
L858R	*n* = 6	*n* = 191		*n* = 6	*n* = 36	
PFS	11.2 (11.3)	4.1 (5.2)	0.070	11.3 (11.3)	4.4 (4.8)	0.102
OSpTKI	36.9 (29.4)	12.6 (20.2)	0.008	36.9 (29.4)	11.8 (32.9)	0.050

Abbreviations: AICT, anti‐angiogenesis plus immunochemotherapy; CT, chemotherapy; Gem, gemcitabine; IO, immunotherapy; IQR, interquartile range; Pem, pemetrexed; PFS, progression‐free survival; PS, propensity score; Tax, taxanes; TKI, tyrosine kinase inhibitor.

^a^ Comparison of survival between patients receiving AICT and those not receiving AICT using the Mann–Whitney *U* test.

**TABLE 3:** Multivariable Cox proportional hazards regression analysis for the survival of the enrolled patients and different subgroups.

Survival	Original cohort (*n* = 412)			PS‐matched cohort (*n* = 151)		
	HR	95% CI	*p* ^a^	HR	95% CI	*p* ^b^
Original						
PFS	0.59	0.39–0.90	0.014	0.56	0.35–0.89	0.014
OSpTKI	0.41	0.24–0.69	< 0.001	0.45	0.26–0.79	0.006
By PD‐L1						
≥ 1%						
PFS	0.43	0.23–0.81	0.009	0.35	0.18–0.70	0.003
OSpTKI	0.46	0.20–1.04	0.062	0.49	0.22–1.13	0.095
< 1%						
PFS	0.77	0.43–1.36	0.280	0.89	0.47–1.67	0.709
OSpTKI	0.36	0.18–0.72	0.004	0.41	0.20–0.86	0.018
By AICT treatment lines						
2 L						
PFS	0.44	0.22–0.89	0.022	0.39	0.18–0.83	0.015
OSpTKI	0.35	0.13–0.94	0.038	0.44	0.16–1.19	0.106
> 2 L						
PFS	0.72	0.42–1.22	0.221	0.74	0.41–1.34	0.318
OSpTKI	0.43	0.24–0.80	0.008	0.46	0.24–0.90	0.022
By AICT regimen						
CT with pemetrexed						
PFS	0.48	0.27–0.85	0.012	0.42	0.23–0.78	0.006
OSpTKI	0.38	0.18–0.82	0.014	0.44	0.20–0.97	0.043
CT with gemcitabine or taxane						
PFS	0.86	0.45–1.66	0.659	1.04	0.53–2.05	0.914
OSpTKI	0.39	0.18–0.84	0.016	0.43	0.20–0.95	0.037
Other IO and CT						
PFS	0.50	0.12–2.03	0.329	0.42	0.10–1.71	0.223
OSpTKI	0.66	0.16–2.72	0.565	0.62	0.15–2.55	0.508
By EGFR mutation						
Exon 19 deletion						
PFS	0.41	0.19–0.88	0.022	0.60	0.34–1.08	0.086
OSpTKI	0.52	0.19–1.44	0.209	0.56	0.28–1.11	0.096
L858R						
PFS	0.40	0.13–1.29	0.125	0.42	0.15–1.17	0.098
OSpTKI	0.39	0.10–1.63	0.199	0.27	0.07–1.05	0.060

^a^ Adjusted variables including age, gender, ECOG‐PS, stage, histology, distant metastatic site, EGFR mutant subtypes, frontline EGFR‐TKI.

^b^ Adjusted variables including frontline EGFR‐TKI.

In the PS‐matched cohort, the analysis indicated that patients receiving AICT had a median PFS of 6.2 versus 3.7 months in the non‐AICT group (*p* = 0.012) and a median OSpTKI of 17.7 months as opposed to 10.8 months (*p* = 0.033), as shown in Table 2. Kaplan–Meier curves reflected these trends, with statistically significant differences in PFS and OSpTKI (log‐rank *p* = 0.031 and 0.030, respectively) as illustrated in Figure 2C,D. Multivariable Cox proportional hazards regression provided evidence of a correlation between AICT and improved survival outcomes (PFS: HR = 0.56, 95% CI = 0.35–0.89, *p* = 0.014; OSpTKI: HR = 0.45, 95% CI = 0.26–0.79, *p* = 0.006) (Table 3).

### 3.3 Survival Analysis for Different Subgroups

**FIGURE 3:**
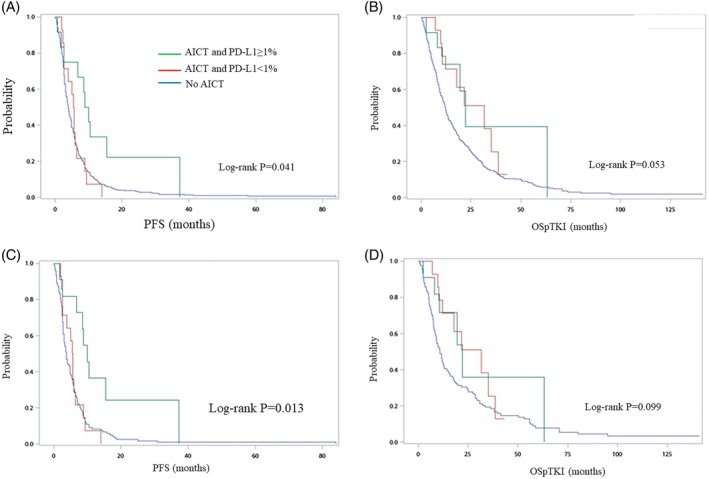
Kaplan–Meier plot and log‐rank test for survival in patients receiving AICT and the control group according to PD‐L1 levels. For the full cohort (A) PFS, (B) OSpTKI. For the PS‐matched cohort (C) PFS, (D) OSpTKI. AICT, anti‐angiogenesis plus immunochemotherapy; Gem, gemcitabine; IO, immunotherapy; OSpTKI, overall survival post‐frontline tyrosine kinase inhibitor; Pem, pemetrexed; PFS, progression‐free survival; PS, propensity score; Tax, taxanes.

After stratifying patients by PD‐L1 expression levels, we found that patients who received AICT had an increase in PFS compared to those who did not receive AICT for PD‐L1 ≥ 1% (9.4 vs. 3.9 months, *p* = 0.017), but not for patients with PD‐L1 < 1% (5.6 vs. 3.9 months, *p* = 0.315) (Table 2). These results in PFS remained consistent after propensity score matching. Similarly, the Kaplan–Meier curves showed significantly improved survival for PD‐L1 ≥ 1% (log‐rank *p* = 0.013 for the original cohort and log‐rank *p* = 0.004 for the PS‐matched cohort), but not for PD‐L1 < 1% (Figure 3). Multivariable COX regression analyses also demonstrated increased PFS (HR = 0.43, 95% CI = 0.23–0.81, *p* = 0.009 for the original cohort, and HR = 0.35, 95% CI = 0.18–0.70, *p* = 0.003 for the PS‐matched cohort) (Table 3). However, there was no statistically significant difference in OSpTKI regardless of PD‐L1 expression level for patients receiving AICT (Table 2). There was a trend of increased OSpTKI in patients receiving AICT regardless of PD‐L1 level in both the original cohort and PS‐matched cohort as shown by the Kaplan–Meier curves (Figure 3) and the multivariable COX regression model (Table 3).

After stratifying patients according to the treatment line of AICT, we observed an increased trend in PFS among patients who received AICT as a second‐line treatment, although this observation did not manifest as a definitive survival advantage according to Table 2. Additionally, the Kaplan–Meier curves indicated a trend toward extended PFS and OSpTKI in patients treated with AICT as a second‐line therapy, a pattern not observed in treatments administered beyond the second line (Figure S2). Cox regression analysis demonstrated a correlation between AICT as a second‐line treatment and an increase in PFS for both the original and PS‐matched cohorts, with a notable enhancement in OSpTKI within the original cohort, as reported in Table 3. The application of AICT beyond the second line did not result in a significant rise in PFS, despite its association with an increase in OSpTKI. No survival differences were discerned between the use of AICT as a second‐line therapy and its application in subsequent lines within either the original or PS‐matched cohorts, by Kaplan–Meier curves and Cox regression analysis.

After stratification of patients by AICT treatment regimen, it was observed that incorporating pemetrexed into the chemotherapy regimen correlated with an improvement in both PFS and OSpTKI, as shown in Table 3. Conversely, the use of taxanes or gemcitabine was linked to an enhancement in OSpTKI, albeit without a significant impact on PFS. The variation in immunotherapy regimens showed no significant impact on survival outcomes, likely due to limited statistical power from a small sample size of only two cases in this cohort. The Kaplan–Meier analysis for the PS‐matched cohort revealed that using pemetrexed correlated with enhanced PFS. Conversely, no significant survival benefit was observed with the use of other immunotherapies, taxanes, or gemcitabine in the treatment regimen (Figure S3) After stratifying patients by EGFR mutation type, we found a trend of increased PFS and OS in patients with Del19 or L858R mutations (Table 3, Figure S4).

## 4 Discussion

After analyzing a retrospective cohort of 412 patients from NCKUH, we found that AICT is associated with significantly improved PFS and OSpTKI. This association was evident in both the original cohort and propensity score‐matched subset, underscoring the potential survival benefits of AICT, particularly in specific patient subgroups when used as a second‐line treatment or in combination with pemetrexed. While the use of AICT beyond the second line showed a correlation with PFS, it did not significantly impact OSpTKI, highlighting the importance of treatment selection and timely treatment in optimizing patient outcomes. These results contribute valuable insights into the role of AICT in the treatment of EGFR‐mutated lung adenocarcinoma, suggesting a potential paradigm shift in the management of this challenging patient population.

In our cohort, the median PFS and OSpTKI were 5.9 and 17.9 months, respectively, which are consistent with previous real‐world studies [19, 20]. We also observed a PFS benefit, but not an OSpTKI benefit, for patients who received AICT compared to those who did not receive AICT after disease progression from frontline EGFR‐TKI treatment. These results align with the EGFR mutant subgroup analyses from the IMPOWER 150 study [10, 21], which demonstrated a PFS benefit for EGFR mutant patients following the failure of EGFR‐TKI treatment with atezolizumab, bevacizumab, and chemotherapy.

There seems to be a synergistic effect between immune checkpoint inhibitors and anti‐angiogenic agents. In the view of pathophysiology, pro‐angiogenic factors have been found to affect the maturation and function of immune cells. For example, VEGF interferes with the maturation of dendritic cells, thereby suppressing T‐cell priming and inducing exhaustion of CD8+ T cells [22]. Additionally, immune cells in the tumor microenvironment, such as M2‐like macrophages, TH2 cells, and Treg cells, secrete pro‐angiogenic factors that promote angiogenesis, leading to the formation of disorganized vascularity in the tumor, which further prevents CD8+ T‐cell trafficking into the tumor microenvironment [22, 23]. Conversely, by secreting IFN‐γ, CD8+ T cells and CD4+ TH1 cells suppress angiogenesis and promote vascular maturation [22]. Clinically, using immune checkpoint inhibitors or anti‐angiogenic agents alone with chemotherapy did not provide a survival benefit for NSCLC patients after TKI treatment failure. Two recently published large randomized clinical trials, Keynote‐789 and Checkmate‐722, which treated post‐TKI treatment failure NSCLC patients with pembrolizumab or nivolumab and platinum‐based doublet chemotherapy, did not show PFS or OS benefits [8, 9]. In a post hoc analysis of the IMPOWER 150 study, the ABCP regimen showed a PFS benefit over the ACP (atezolizumab, carboplatin, and paclitaxel) or BCP (bevacizumab, carboplatin, and paclitaxel) regimens [21]. These results emphasize the necessity of combining immune checkpoint inhibitors with anti‐angiogenic agents for these patients.

We found that patients with PD‐L1 ≥ 1% had superior PFS when receiving AICT treatment compared to non‐AICT regimens. In contrast, patients with PD‐L1 < 1% did not benefit from AICT treatment. Our results are consistent with previous prospective randomized trials. Specifically, in the subgroup analysis of the ATTLAS trial, patients with PD‐L1 ≥ 1% showed improved PFS with the ABCP regimen, but this was not observed in patients with PD‐L1 < 1% [11]. This suggests that the combination of immune checkpoint inhibitors and anti‐angiogenic agents does not benefit patients with EGFR mutations if there is no PD‐L1 expression. However, this combination might bring benefits for patients with PD‐L1 ≥ 1%.

In our subgroup analysis, patients who received pemetrexed as part of their chemotherapy doublet had superior PFS in both COX regression and Kaplan–Meier analyses. To the best of our knowledge, there has been no study directly comparing pemetrexed with other chemotherapies in combination with immune therapy and anti‐angiogenic agents. However, some single‐arm studies have explored the benefit of pemetrexed in such combinations. For instance, Lam et al. reported a median PFS of 9.4 months and a 1‐year OS rate of 72.5% in 40 NSCLC patients treated with a combination of atezolizumab, bevacizumab, pemetrexed, and carboplatin after EGFR‐TKI treatment failure [24]. In comparison, Watanabe et al. treated 60 non‐squamous NSCLC patients harboring sensitizing EGFR mutations with the ABCP regimen after frontline EGFR‐TKI, resulting in a median PFS of 7.4 months and an OS of 23.1 months in the cohort [25]. Some preclinical studies have shown that pemetrexed may enhance the effect of anti‐PD‐L1 treatment. Cavazzoni et al. demonstrated that pemetrexed increases PD‐L1 expression in NSCLC and potentiates the effects of anti‐PD‐L1 immunotherapy [26]. Lu et al. also found that the thymidylate synthase inhibition by pemetrexed leads to the activation of ROS and NF‐κB, further increasing PD‐L1 expression and potentially enhancing the benefits of AICT [27]. Further investigation is needed to determine the survival benefits of AICT with different chemotherapy regimens.

While this study sheds light on the potential benefits of AICT for patients with EGFR‐mutated lung adenocarcinoma, it comes with several limitations that must be acknowledged. Firstly, the retrospective nature and single‐center scope of the research, along with a limited number of participants, may affect the broad applicability and strength of our conclusions. Prospective, multicenter studies would be valuable to validate and extend our findings. Secondly, the heterogeneity in treatment lines and AICT regimens within our cohort could potentially impact the study's results. To address this, we conducted several analyses, including multivariable analysis and propensity score matching, both in the original cohort and within subgroups defined by different treatment lines and AICT regimens. Thirdly, since no patient in our cohort had a PD‐L1 expression of ≥ 50%, we categorized enrolled patients using the 1% PD‐L1 cutoff value. The effectiveness of AICT for patients with PD‐L1 ≥ 50% in our cohort is unknown. Fourth, the timing of PD‐L1 level assessment was not consistent for all patients who received AICT. In our cohort, most patients had their PD‐L1 levels assessed using specimens from the initial diagnosis, rather than specimens obtained just prior to AICT treatment. However, the majority of patients did not change PD‐L1 level categories, unless they had received immunotherapy [28]. In our study, no PD‐L1 levels were retrieved from specimens after the administration of immunotherapy, and therefore, we believe the PD‐L1 categorization is reliable. Fifth, radiotherapy may enhance the immunogenicity of the tumor microenvironment and could potentially improve the efficacy of immunotherapy. Including both radiotherapy and PD‐L1 levels could provide more valuable insights for predicting the effect of immunotherapy. However, due to the small sample size, we were unable to further divide the patients based on both PD‐L1 levels and radiotherapy.

In conclusion, this study provides real‐world evidence that AICT improves PFS and OSpTKI in patients with advanced‐stage EGFR‐mutated NSCLC following progression on frontline EGFR‐TKI therapies. These findings support the integration of AICT into the treatment regimen for selected patient subgroups, particularly for patients with PD‐L1 ≥ 1%, as a second‐line therapy or in combination with pemetrexed, to optimize survival outcomes. Future research should focus on personalizing treatment strategies to enhance efficacy and quality of life for patients with this challenging disease.

## Conflicts of Interest

The authors declare no conflicts of interest.

## Supporting information


**Figure S1.** Illustration for survivals. (A) PFSpTKI, (B) PFSwAICT, (C) OSpTKI.
**Figure S2.** Kaplan–Meier plot and log‐rank test for survival in the AICT use group stratified by different treatment lines, compared with the control group. For full cohort (A) PFS, (B) OSpTKI. For PS‐matched cohort (C) PFS, (D) OSpTKI. AICT, anti‐angiogenesis plus immunochemotherapy; OSpTKI, overall survival post‐frontline tyrosine kinase inhibitor; PFS, progression‐free survival; PS, propensity score.
**Figure S3.** Kaplan–Meier plot and log‐rank test for survival in the AICT use group stratified by treatment regimen, compared with the control group. For full cohort (A) PFS, (B) OSpTKI. For PS‐matched cohort (C) PFS, (D) OSpTKI. AICT, anti‐angiogenesis plus immunochemotherapy; Gem, gemcitabine; IO, immunotherapy; OSpTKI, overall survival post‐frontline tyrosine kinase inhibitor; Pem, pemetrexed; PFS, progression‐free survival; PS, propensity score; Tax, taxanes.
Figure S4.

## Data Availability

The data that support the findings of this study are available on request from the corresponding author. The data are not publicly available due to privacy or ethical restrictions.
