# Supplementary material for: Comparative Survival Analysis of Anti‐Angiogenic Agent Plus Immunochemotherapy in NSCLC Patients After Frontline EGFR‐TKI Treatment: A Retrospective Cohort Study
Source: Kaohsiung J Med Sci. 2025 Apr 28;41(7):e70023. doi: 10.1002/kjm2.70023 (PMC12245087; doi:10.1002/kjm2.70023)
Supplement: Supplementary file 1 — Figure S1. Illustration for survivals. (A) PFSpTKI, (B) PFSwAICT, (C) OSpTKI. Figure S2. Kaplan–Meier plot and log‐rank test for survival in the AICT use group stratified by different treatment lines, compared with the control group. For full cohort (A) PFS, (B) OSpTKI. For PS‐matched cohort (C) PFS, (D) OSpTKI. AICT, anti‐angiogenesis plus immunochemotherapy; OSpTKI, overall survival post‐frontline tyrosine kinase inhibitor; PFS, progression‐free survival; PS, propensity score. Figure S3. Kaplan–Meier plot and log‐rank test for survival in the AICT use group stratified by treatment regimen, compared with the control group. For full cohort (A) PFS, (B) OSpTKI. For PS‐matched cohort (C) PFS, (D) OSpTKI. AICT, anti‐angiogenesis plus immunochemotherapy; Gem, gemcitabine; IO, immunotherapy; OSpTKI, overall survival post‐frontline tyrosine kinase inhibitor; Pem, pemetrexed; PFS, progression‐free survival; PS, propensity score; Tax, taxanes. Figure S4. [file KJM2-41-e70023-s001.docx]

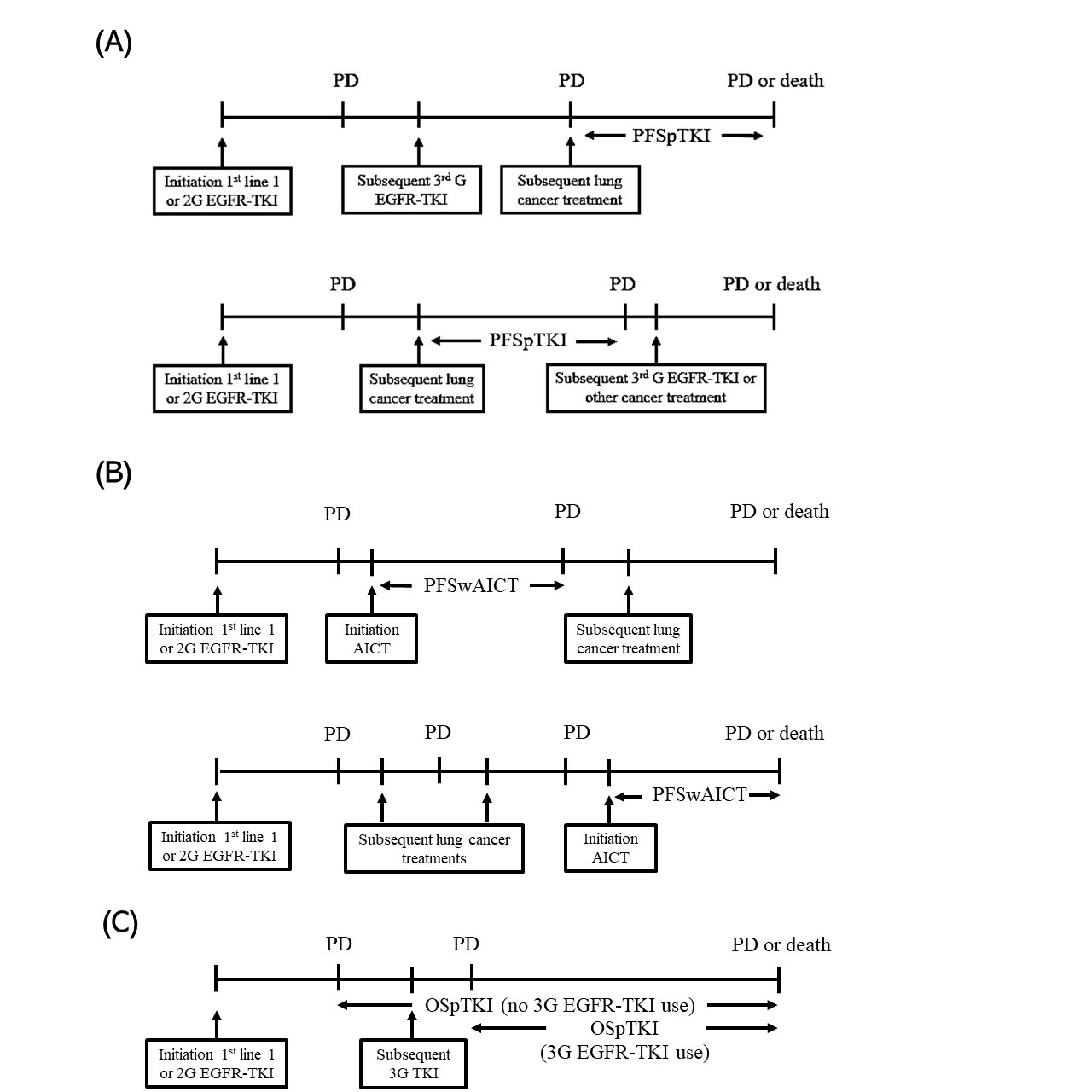


**Supplementary figure 1. Illustration for survivals.** (A) PFSpTKI, (B) PFSwAICT, (C) OSpTKI.


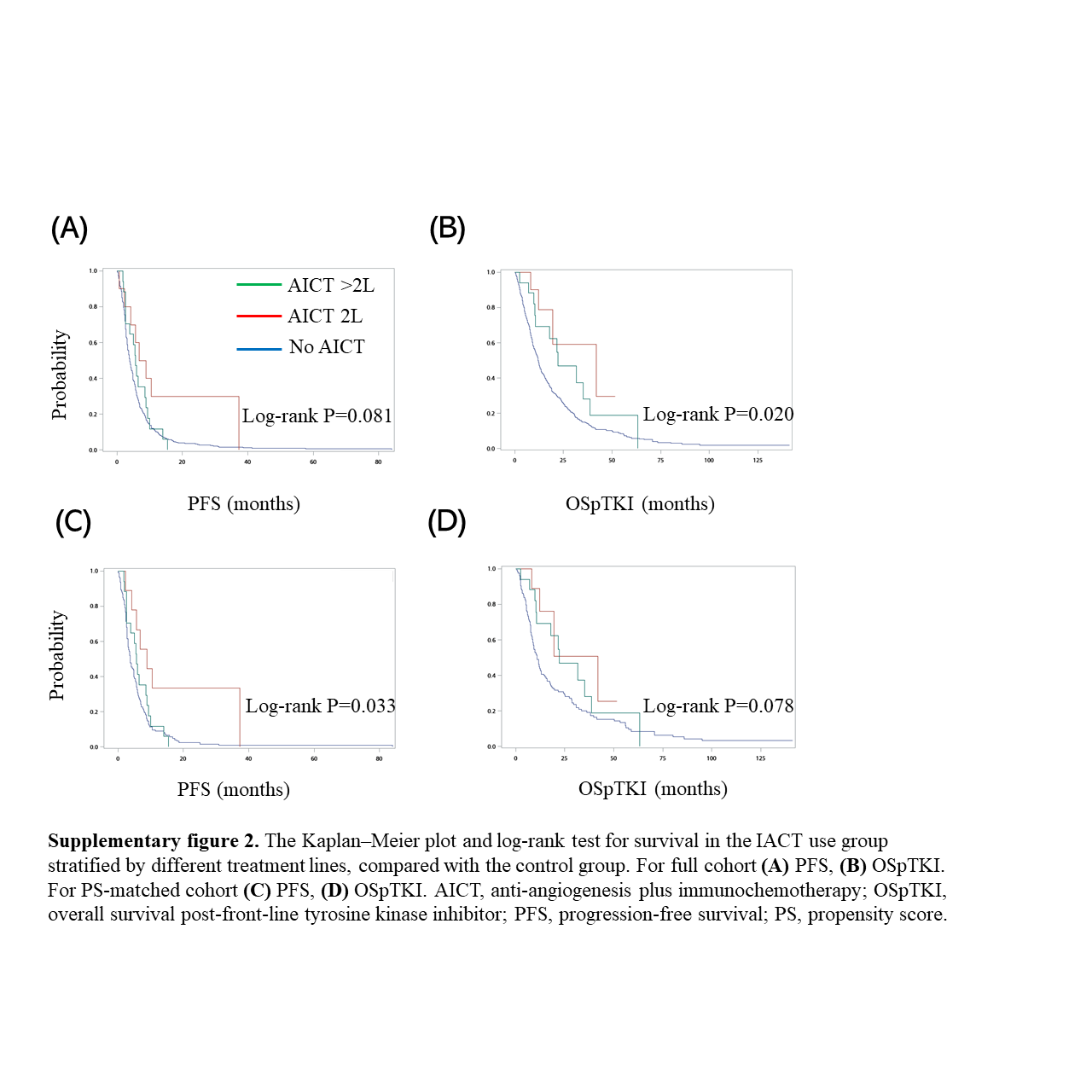


**Supplementary figure 2.** The Kaplan–Meier plot and log-rank test for survival in the IACT use group stratified by different treatment lines, compared with the control group. For full cohort **(A)** PFS, **(B)** OSpTKI. For PS-matched cohort **(C)** PFS, **(D)** OSpTKI. AICT, anti-angiogenesis plus immunochemotherapy; OSpTKI, overall survival post-front-line tyrosine kinase inhibitor; PFS, progression-free survival; PS, propensity score.


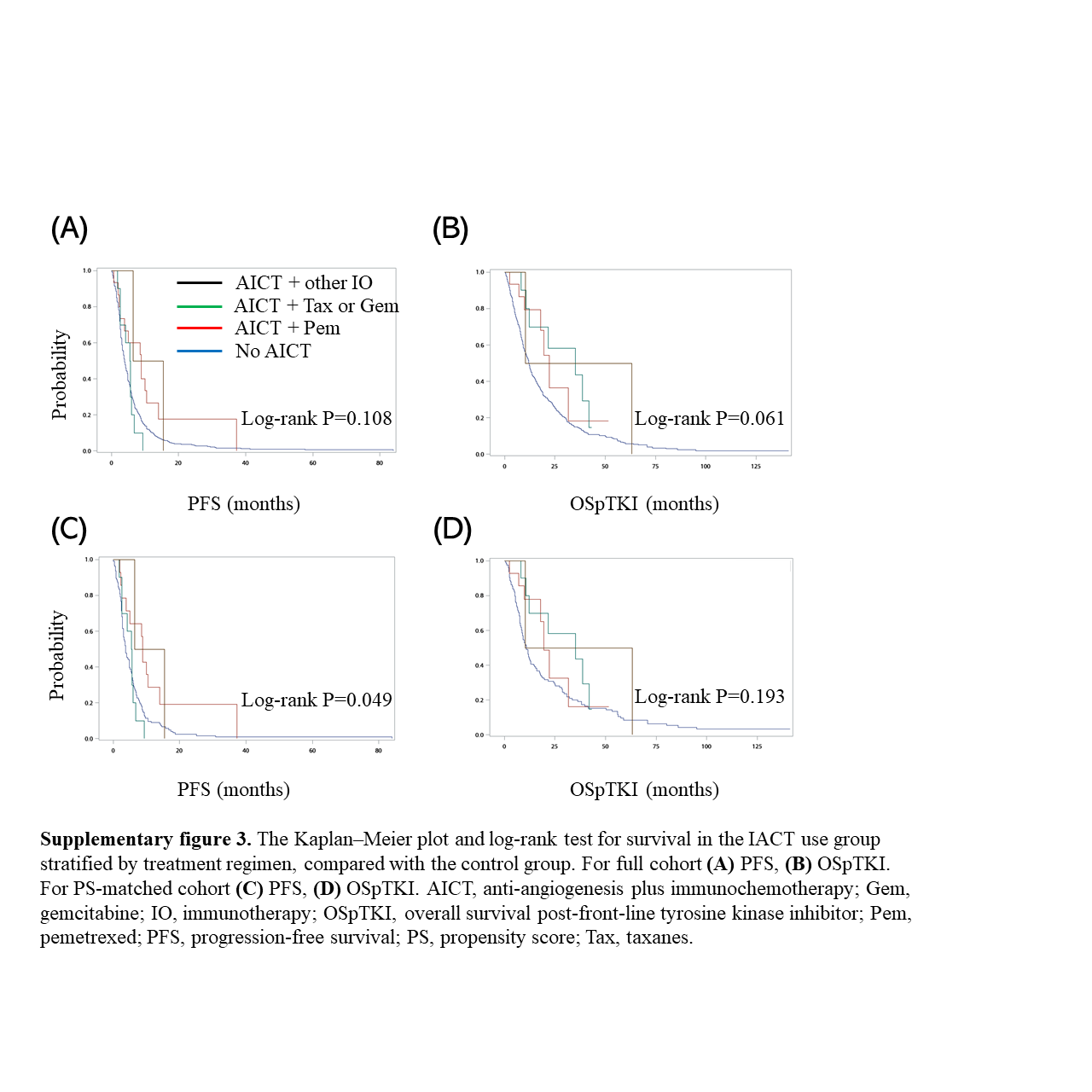


**Supplementary figure 3.** The Kaplan–Meier plot and log-rank test for survival in the IACT use group stratified by treatment regimen, compared with the control group. For full cohort **(A)** PFS, **(B)** OSpTKI. For PS-matched cohort **(C)** PFS, **(D)** OSpTKI. AICT, anti-angiogenesis plus immunochemotherapy; Gem, gemcitabine; IO, immunotherapy; OSpTKI, overall survival post-front-line tyrosine kinase inhibitor; Pem, pemetrexed; PFS, progression-free survival; PS, propensity score; Tax, taxanes.


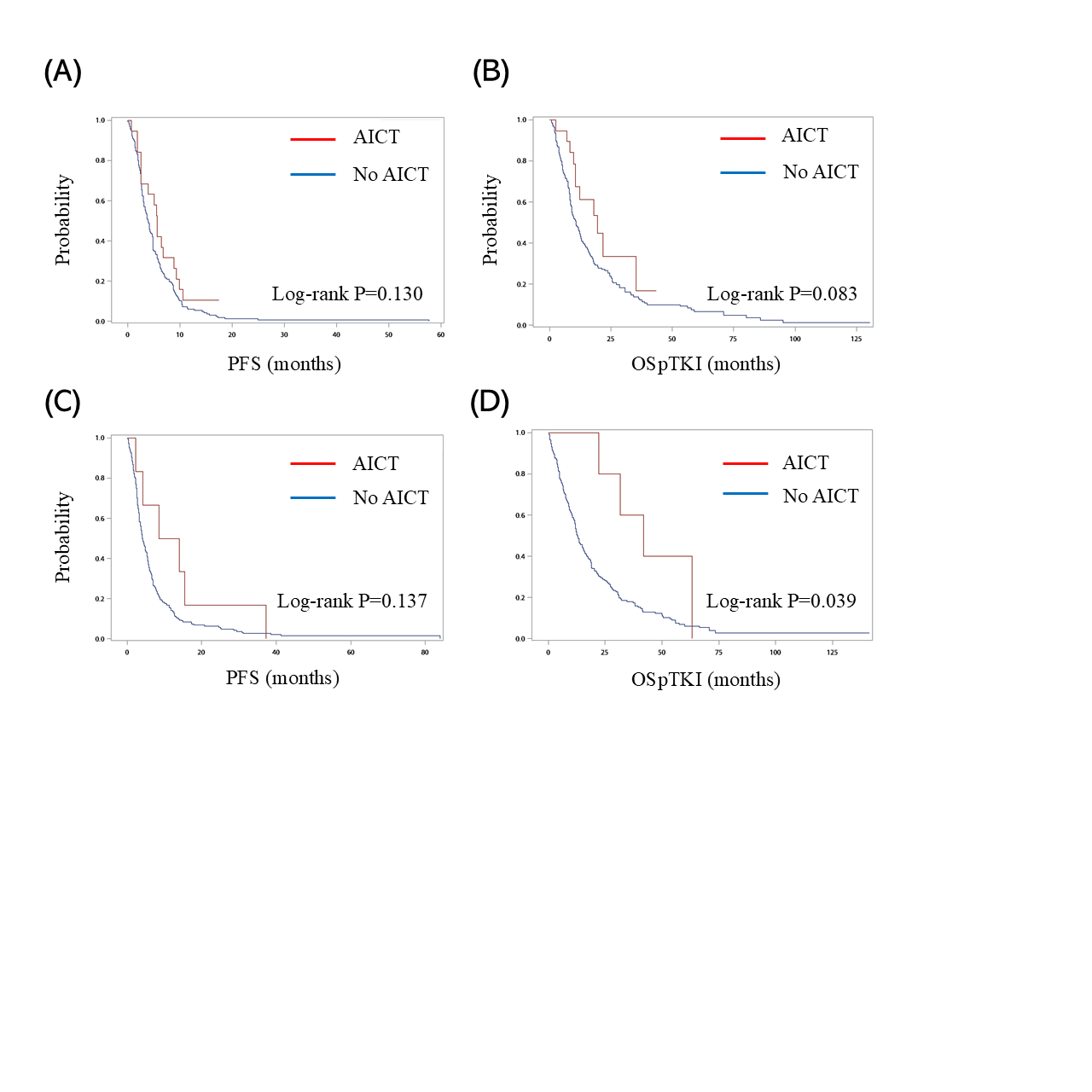


**Supplementary figure 4.** The Kaplan–Meier plot and log-rank test for survival in the IACT use group stratified by EGFR mutation types, compared with the control group. For Exon 19 deletion cohort **(A)** PFS, **(B)** OSpTKI. For L858R cohort **(C)** PFS, **(D)** OSpTKI. AICT, anti-angiogenesis plus immunochemotherapy; OSpTKI, overall survival post-front-line tyrosine kinase inhibitor; PFS, progression-free survival.
